# Neutrophil extracellular traps in tumor progression and immunotherapy

**DOI:** 10.3389/fimmu.2023.1135086

**Published:** 2023-03-13

**Authors:** Meina Yan, Yifeng Gu, Hongxia Sun, Qinghong Ge

**Affiliations:** ^1^ Department of Laboratory Medicine, The Affiliated Suzhou Hospital of Nanjing Medical University, Suzhou Municipal Hospital, Gusu School, Nanjing Medical University, Suzhou, Jiangsu, China; ^2^ Department of Laboratory Medicine, Tumor Hospital Affiliated to Nantong University, Nantong, Jiangsu, China; ^3^ Department of Gynecology and Obstetrics, The Affiliated Suzhou Hospital of Nanjing Medical University, Suzhou Municipal Hospital, Gusu School, Nanjing Medical University, Suzhou, Jiangsu, China

**Keywords:** neutrophil extracellular traps, anti-tumor immunity, immunotherapy, tumor microenvironment, tumor progression

## Abstract

Tumor immunity is a growing field of research that involves immune cells within the tumor microenvironment. Neutrophil extracellular traps (NETs) are neutrophil-derived extracellular web-like chromatin structures that are composed of histones and granule proteins. Initially discovered as the predominant host defense against pathogens, NETs have attracted increasing attention due to they have also been tightly associated with tumor. Excessive NET formation has been linked to increased tumor growth, metastasis, and drug resistance. Moreover, through direct and/or indirect effects on immune cells, an abnormal increase in NETs benefits immune exclusion and inhibits T-cell mediated antitumor immune responses. In this review, we summarize the recent but rapid progress in understanding the pivotal roles of NETs in tumor and anti-tumor immunity, highlighting the most relevant challenges in the field. We believe that NETs may be a promising therapeutic target for tumor immunotherapy.

## 1 Introduction

Recent studies have shown that strategies that increase anti-tumor immune responses play important roles in the fight against cancer (1, 2). Although neutrophils are the first line of defense in innate immunity, tumour-associated neutrophils (TANs) could promote tumor progression (3). Moreover, under certain circumstances, the tumor microenvironment (TME) can attract neutrophils to tumor tissue and functionally modulate them to release web-like structures to form neutrophil extracellular traps (NETs) (4). NETs are composed of DNA fragments coated with histones and toxic granule proteins, such as citrullinated histone H3 (H3Cit), myeloperoxidase (MPO), neutrophil elastase (NE), cathepsin G (CG), matrix metalloproteinase 9 (MMP-9), which were first discovered by Volker Brinkmann (5). NETs can capture and kill pathogens such as bacteria (6), fungi (7), viruses (8) and parasites (9). However, dysregulated NETs are harmful to the host.

Extensive studies have confirmed that uncontrolled and excessive NETs are involved in the pathogenesis of autoimmune disease (10, 11), cardiovascular disease (12), inflammatory disease (13) and cancer (14). It is worth mentioning that the function of NETs in tumors is gradually expanding. NETs are related to detrimental outcomes in breast cancer, pancreatic cancer, and hepatocellular carcinoma (15–17). NETs can promote tumor growth, invasion, metastasis, and drug resistance (18–20). Although accumulating evidence has clarified how NETs contribute to tumor progression, the role of NETs in anti-tumor immune responses is less clear. Therefore, further studies are necessary to elucidate the effects of NETs on tumor immunity. This review primarily focuses on the function of NETs in tumor and anti-tumor immunity, and highlights their application in tumor immunotherapy.

## 2 NET structure and formation

**Figure 1:**
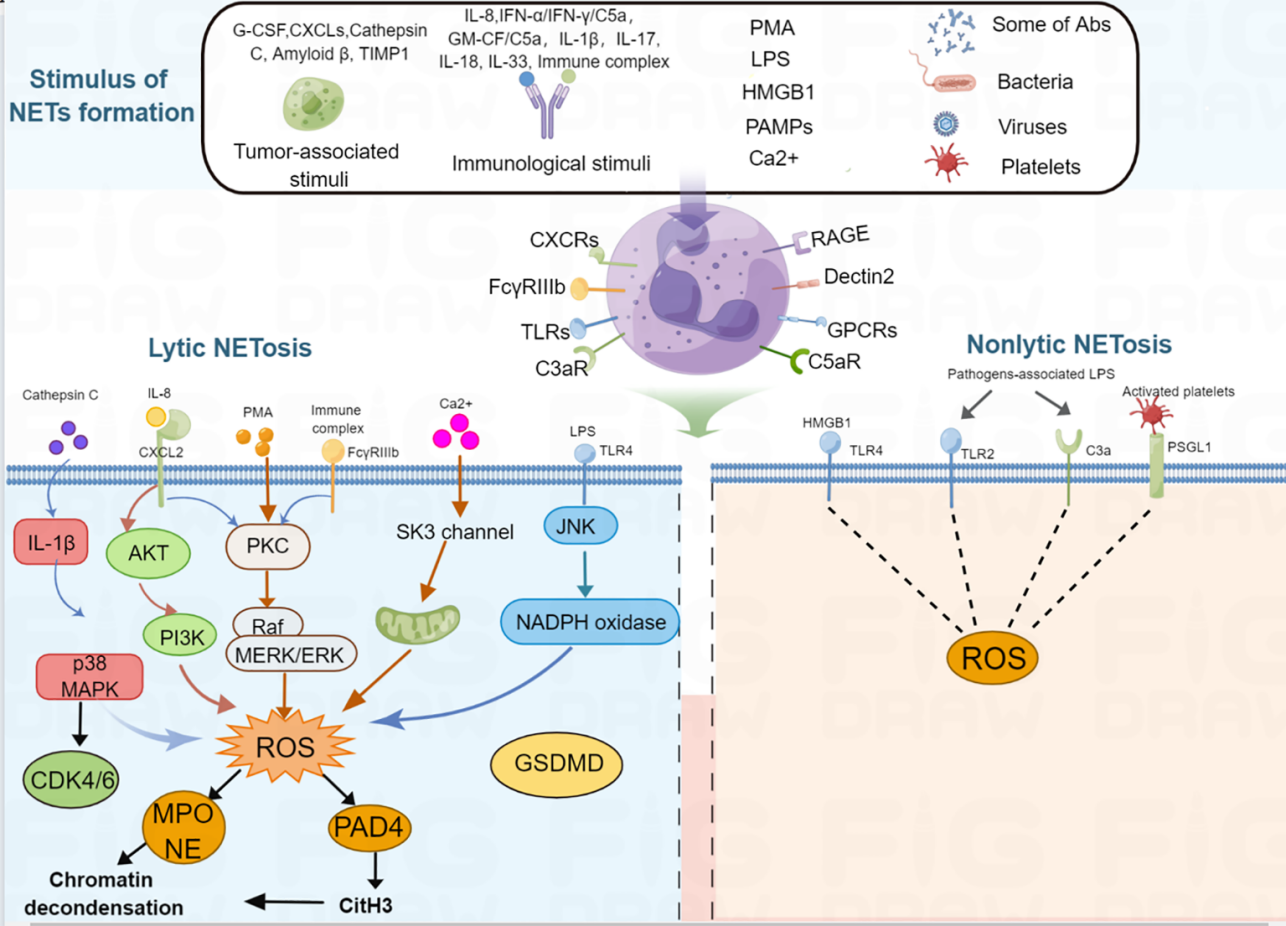
Schematic representation of NET formation. Different stimuli, such as PMA, tumor-associated stimuli, immunological stimuli, IL-1β, IL-17, IL-18, IL-33, LPS, PAMPs, some antibodies, activated platelets, bacteria, viruses, Ca2+ can induce NET formation. For lytic NETosis, external stimuli produce different kinds of ROS-inducing receptors, activating neutrophils to produce intracellular ROS, ROS further activates MPO and PAD4, then MPO activates NE and PAD4 citrullinates H3, therefore, leads to nuclear envelope disintegration, chromatin decondensation, cell membrane breakdown, NET formation. For non-lytic NETosis, some stimuli, such as *Staphylococcus aureus* and *Candida albicans*-associated LPS and HMGB1 can induce NET formation through a non-lytic manner.

NETs are large, extracellular, web-like structures composed of DNA fibers coated with histones and granule proteins. Various stimuli trigger NET formation, such as lipopolysaccharides (LPS), phorbol 12-myristate 13-acetate (PMA) (5), high mobility group box 1 [HMGB1] (21), tumor-associated stimuli (tumor-associated antigen, granulocyte-colony stimulating factor [G-CSF] (22), C-X-C motif chemokine ligands [CXCLs] (23), cathepsin C (24), amyloid β (18), tissue inhibitor of metalloproteinases-1 [TIMP1] (16)), different immunological stimuli (interleukin [IL]-8/CXCL8, interferon [IFN]-α/IFN-γ/C5a, granulocyte-macrophage [GM-CSF/C5a), IL-1β, IL-17, IL-18, IL-33, immune complex (5, 20, 25–30), and other pathogen-associated molecular pattern molecules(PAMPs) (31, 32), autoantibodies (33), activated platelets (34), bacteria (35, 36), viruses (37), fungi, calcium ionophores (38), cigarette smoke (39), free fatty acids (40), and bleomyci (41) (**Table 1**). These stimuli activate the cell surface receptors of neutrophils; for example, HMGB1 recognizes advanced glycation end products (RAGE) receptor and toll-like receptor 4 (TLR4) (42), C3a recognizes C3a receptor (C3aR) (43), C5a recognizes C5a receptor (C5aR) (44), CXC chemokines recognize CXC chemokine receptors (CXCRs) (23), immune complex activate the FcγRIIIb receptor (45), LPS and platelets activate the toll-like receptor (TLR) (46, 47), bacterial products recognize G protein-coupled receptors (48), fungi recognize the Dectin1 and Dectin 2 receptor (49, 50). After the stimuli activate the receptors of the neutrophils, different intracellular signaling mechanisms are further activated, leading to the formation of two types of NETs. The classical form is lytic NETosis, which is considered a type of slow cell death. Besides, this process depends on the NADPH oxidase-mediated generation of reactive oxygen species (ROS), as evidenced by chronic granulomatous disease patients with mutations in the NADPH oxidase that fail to form NETs (51). Many reactive oxygen species (ROS)-inducing factors, including PMA, C5a, LPS, TLR-4, immune complexes, IL-8, cathepsin C, calcium ionophores activate NOX *via* different molecular pathways that cause ROS generation (24, 25, 30, 52–55). Accumulation of ROS triggers the escape of MPO and NE from the granules (56). MPO first activates NE to degrade the cytoskeleton in the cytoplasm (57). Subsequently, NE translates to the nucleus to cleave histones that contributes to chromatin decondensation (56). Blocking NE by NE inhibitor or serum leukocyte protease inhibitor (SLPI) disrupts NET formation (56), suggest that NE is required for chromatin extrusion. Moreover, in the late stage of chromatin decondensation, MPO binds to chromatin to promote further decondensation (56). In parallel, ROS synthesis also leads to the activation of peptidyl arginine deiminase 4 (PAD4), a calcium-dependent enzyme, which catalyzes histone citrullination, thereby promoting chromatin decondensation (58). Further study showed that inhibition of PAD4 *in vitro* greatly reduced the process of NETosis, and PAD4 knockout mice failed to produce NETs *in vivo*, indicated that PAD4 is critical for NET formation (6). Recently, Amulic et al., have added on another critical step in NET formation: the activation of cyclin-dependent kinases (CDKs) 4 and 6 (59). Although the mechanism is still unclear, this study suggested CDK4/6 likely function downstream of MAPK and ROS, and CDK6 is required, while CDK4 is partially required for NET formation (59, 60). Finally, nuclear membrane breakage, nuclear DNA and proteins are released. Released DNA further decorated with NE, MPO and cytosolic proteins, followed by plasma membrane rupture and NET extrusion and eventually lysis (56, 58). Besides, there are also noncanonical signaling triggers NET formation independently of ROS and PAD4, which mediated by a pore-forming protein gasdermin D (GSDMD) (36, 61). The second type of NET is a non-cell-death form in which NET are rapidly released from live cells without nuclear membrane disruption or loss of membrane disruption, which accompanied by granule proteins; this is known as nonlytic NET formation (25, 32, 34, 62). In this process, NETs were also found to include mitochondria DNA (mtDNA) when neutrophils are stimulated with LPS or C5a (25). Besides, it has been confirmed that some pathogens, such as S. aureus and C. albicans induce a rapid nonlytic NET formation by activating TLR2 and C3 (62). Moreover, this type of nonlytic NET formation is critical to acute invasive infection (62). Additionally, LPS-stimulated platelets could also promote nonlytic NETosis by activating platelet TLR4 (31, 34). However, the molecular mechanisms of nonlytic NETosis are still poorly understood. It can be ROS dependent or independent. A summary of NETosis induced by various stimuli is shown in **Figure 1**.

**Table 1:** Stimuli that induce NET formation.

Stimuli	References
LPS	(5)
PMA	(5)
HMGB1	(21)
G-CSF	(22)
CXCLs	(23)
Cathepsin C	(24)
Amyloid β	(18)
TIMP1	(16)
CXCL8/IL-8	(5)
[IFN]-α/IFN-γ/C5a	(25)
GM-CSF/C5a	(25, 26)
IL-1β	(27)
IL-17	(20)
IL-18	(29)
IL-33	(28)
Immune complexes	(30)
Pathogen-associated molecular pattern molecules (PAMPs)	(31, 32)
Autoantibodies	(33)
Activated platelets	(34)
Bacteria	(35, 36)
Viruses	(37)
Fungi	(38)
Calcium ionophores	(38)
Cigarette smoke	(39)
Free fatty acids	(40)
Bleomyci	(41)

Apart from the physiological roles in host defense against pathogens, uncontrolled NET formation has been found to play a pivotal role in atherosclerosis (63, 64), coronary artery disease (65), autoimmune disease (66, 67), sepsis (68), metabolic disease (69), coronavirus disease 19 (COVID-19) (37, 70), and cancer (71).

## 3 Evidence of NETs promoting tumor progression

Accumulating evidence suggests that the TME can induce NET formation in various types of cancer, including hematologic malignancy (72–74) and solid tumors, such as breast cancer (75), ovarian cancer (76), gastric cancer (77), hepatic carcinoma (78), lung cancer (79), and colon cancer (80, 81). In particular, studies have revealed that NETs are increased in the peripheral blood and tumor tissues of patients with cancer (16, 76, 82). To date, NET formation in tumors may be partly due to tumor cells interacting directly and indirectly with neutrophils *via* the production of cytokines, chemokines, proteases, extracellular vesicles. Recent studies have shown that NETs can promote tumor progression *via* different mechanisms (**Table 2**).

**Table 2:** The roles of NETs in the cancer progression.

Cancer type	Detected NETs marker	NETs Formation Mechanism	Relevance to cancer progression	Potential Mechanisms	Ref.
Hepatocellular carcinoma (HCC)	MPO-DNA/H3cit	Cancer cell-derived IL-8	Promote tumor invasiveness and metastasis; predict a poor prognosis	Activate TLR4/9-COX2; increase cathepsin G; oxidize mtDNA	(77, 83, 84)
Breast cancer	MPO-DNA/H3cit	Cancer cell-derived cathepsin C	Promote tumor metastasis	Regulate CCDC25-ILK-β-parvin pathway; NF-κB pathway	(15, 24)
Pancreatic cancer	MPO-H3cit	Cancer cell-derived DDR1; TIMP1	Promote cancer cells migration and invasion; promote tumor metastasis; induce immunotherapy resistance	Activate IL-1β/EGFR/ERK pathway; inhibit CD8+ T cell function	(16, 19, 20, 85)
Ovarian cancer	MPO-H3cit	Cancer cell-derived inflammatory factors	Promote tumor metastasis and chemotherapy resistance	Unclear	(75)
Gastric cancer (GC)	MPO-DNA/cfDNA/NE/MPO-H3cit	Cancer cell-derived TME/Postoperative abdominal infectious complication	Promote tumor proliferation, invasion, migration, and metastasis	EMT, Activates TGF-β pathway	(76, 86)
Colon cancer	H3cit	Cancer cell-derived IL-8	Promote cancer proliferation and metastasis	EMT; Releases HMGB1 and activates TLR9 pathways	(79, 80)
Human melanoma	MPO-H3cit	Cancer-associated fibroblasts- derived Amyloid β	Promotes tumor proliferation	Unclear	(18)
Bladder cancer	NE-H3cit	Tumor immune microenvironment-derived HMGB1	Promotes tumor radioresistance	Unclear	(87)
Lung cancer	Unclear	Unclear	Promotes cancer invasion, metastasis	Interaction of TGF-β, IFN-β, and NE-pathways; trap CTCs	(78, 88)
Glioma	MPO-H3cit	IL-8	Promotes tumor proliferation and invasion	HMGB1/RAGE/IL-8 axis	(53)
Acute Promyelocytic Leukaemia (APL)	MPO-DNA/H3cit	Activated platelets	Increases bleeding burden	Damage the integrity of endothelial cells	(71)
Hodgkin Lymphoma	H3cit	Unclear	Correlates with concurrent fibrosis and inflammation	Unclear	(72)
Diffuse large B-cell lymphoma (DLBCL)	MPO-DNA/H3cit	IL-8	Promotes tumor proliferation and migration	TLR9-NFκB-STAT3-p38	(89)
Myeloproliferative neoplasms	H3cit	JAK2	Promotes thrombosis	Unclear	(73)

### 3.1 NETs in tumorigenesis and growth

NETs have been shown to participate in tumor initiation and growth. For instance, non-alcoholic steatohepatitis (NASH) is a risk factor for hepatocellular carcinoma (HCC), and elevated levels of NETs contribute to the progression of NASH to HCC (90). Further study indicated that NASH-associated free fatty acids stimulate NET formation, which increased monocyte-derived macrophages and production of inflammatory cytokines, that contribute to HCC initiation (90). Furthermore, gut-derived LPS induced NET formation through activating TLR4 pathway, which further promoted alcohol-related HCC in mice model (91).Besides, Silvia Guglietta et al., demonstrated that C3aR-dependent NET formation induced protumorigenic neutrophils polarization, and promoted intestinal tumorigenesis (92). Subsequently, in a PAD4 knockout mouse model genetically incapable of NET formation, both subcutaneous tumors and hepatic metastases using murine colorectal (MC38) cells grew significantly more slowly than the WT mice (93). Similarly, human colorectal and hepatocellular cancer cell lines injected subcutaneously in the nu/nu mice treated with DNAse also grew slower (93), suggesting that inhibition of NETosis by preventing NET formation or degrading NETs is correlated with decreased tumor growth *in vivo*. Mechanistically, NETs-associated protein, NE, directly act TLR-4 on the cancer cells, leading to activation of the p38-PGC-1α pathway, followed by increased tumor mitochondrial function and increased tumor growth (93). The direct role of NETs in regulating the metabolism of cancer cells might provide a therapeutic opportunity to effectively halt tumor growth. Another study showed that subcutaneous injection of Lewis lung carcinoma (LLC) cells reduced tumor growth while the B16 melanoma growth was not affected in PAD4-deficient mice (94). Further study showed that G-CSF released from LLC tumor increased more activated CD11b^high^ neutrophils and NETs than B16 tumor, and B16 tumors in WT mice grew faster than the tumors in PAD4-deficient mice after G-CSF treatment (94). This highlights that, different tumors generate different TMEs, which affect the formation of NETs. In addition, it has been reported that increased NETs facilitated cell proliferation and tumor growth in diffuse large B-cell lymphoma (DLBCL) and were correlated with poor prognosis (89). The exact mechanism was that lymphoma cells secreted IL-8 induced NET formation, which depended on the Src and MAPK pathways, in turn, NETs directly activated of the TLR9-NFκB-STAT3-p38 pathway to promote tumor progression (89). In glioma, NETs-derived HMGB1 increased cell proliferation by binding to RAGE and activating the NF-κB signaling pathway (53). Moreover, a recent study demonstrated that DNA released from NETs enhances pancreatic tumor growth (95). And, the mechanism of the pro-tumorigenic effect was not directly through effects on cancer cells, but rather the through NET-DNA induced autophagy-dependent activation of pancreatic stellate cells, causing increased MMP-2 and -9 production to promote cancer progression (95). Hafsa et al. demonstrated that cancer-associated fibroblasts are important factors mediators of NET formation. They found that cancer-associated fibroblast-induced NETs contribute to tumor proliferation in Bladder cancer and pancreatic adenocarcinoma (18). Although further investigation is needed, there is a plenty of *in vitro* and *in vivo* evidence that inhibition of NETs decreased tumor growth in several different cancer types.

### 3.2 NETs in tumor metastasis

Metastasis is a hallmark of advanced stage cancer, which is the primary cause of cancer-related mortality. Moreover, metastasis is a multistep process, including the detachment of cancer cells from the primary tumor, the dissemination of tumor cells to surrounding tissues and distant organs (96). There is also evidence that NETs result in the metastasis cascade of animal and human tumors (97, 98). Epithelial−mesenchymal transition (EMT) is critical for tumor cells to physically disseminate from the primary site, which is the first step in distant metastasis (99). In breast cancer, after treatment with NETs, MCF7 cells gained a migratory and mesenchymal phenotype, accompanied by EMT induction (100). Moreover, the EMT program further upregulated the expression of cancer stem cells (CSCs) markers, such as CD44, and induced a pro-inflammatory response in breast cancer cells (100). These results show that NETs might contribute to breast cancer metastasis through the activation of EMT program. In another study, NETs promoted gastric cancer cells migration through EMT, inhibition of NETs by DNAse-1/GSK-484 upregulated the epithelial marker, E-cadherin, while downregulated the mesenchymal marker (77). Consistently, Jin et al. found that NETs facilitated cell migration and invasion, and EMT in pancreatic cancer. Besides, NETs-mediated EMT is dependent on the activation of IL-1β/EGFR/ERK pathway (85). Following this study, NETs decreased expression of epithelial markers E-cadherin (CDH1), epithelial cell adhesion molecule (EPCAM) and increased expression of mesenchymal markers vimentin (VIM), fibronectin (FN1), which initiates EMT transcriptional programs in colon cancer (80).This EMT-like phenotype increased cell motility and the migration of colorectal cancer cells, which further promoted local invasion and metastasis (80). In non-small cell lung cancer, NETs induced EMT through activating NF‐κB/NLRP3 inflammasome pathway by downregulating the expression of long non-coding RNA MIR503HG, which further enhanced tumor cell metastasis (101). Additionally, one study showed that NETs could induce pancreatic cancer cells migration, invasion and EMT through activating the IL-1β/epidermal growth factor receptor (EGFR)/extracellular signal−regulated kinase (ERK) pathway (85). Taken together, there is increasing evidence that NETs can support tumor metastasis through inducing EMT program. In addition to EMT, NETs also increased cancer cell migration and invasion through other molecular signaling pathways. For example, NET markers, such as MPO-DNA and H3Cit were increased in patients with HCC and predicted a poor prognosis (83). Further studies revealed that NETs-associated Cathepsin G promoted HCC cell invasion through decreasing E-cadherin expression, which promoted HCC metastasis (83). Moreover, HCC cells not only stimulated NET formation, but also modified its composition by increasing the oxidized mitochondrial DNA, which increased HCC cells invasion and lung metastasis *in vitro* and vivo (84). In breast cancer, NETs could promote cell migration and invasion by activating nuclear factor (NF)-κB pathway (75). Another study found that NETs facilitate gastric cancer cell migration, invasion and metastasis by activating the transforming growth factor (TGF)-β pathway (86). Besides, recent research demonstrated that the receptor tyrosine kinase discoid domain receptor 1(DDR1) induces CXCL5 production to recruit neutrophils to stimulate NET formation, leading to pancreatic cancer cell invasion and metastasis (19). Taking into account the above findings, NETs might contribute to metastasis initiation that includes detachment of cancer cells from primary tumor, EMT and increased cell migration and invasion.

Primary cancer cells acquired the migration and invasion ability through EMT or other molecular signaling pathway, then invaded into the surrounding tissues. These cancer cells further intravasate to enter the circulation, where they are termed as circulating tumor cells (CTCs) (96). CTCs must overcome fluid shear stress, immune cells and oxidative stress to colonize distant organs (102). It has been reported that NETs can protect CTCs from cytotoxic immune cells with NETs-mediated physical barrier (103), thus increased metastatic seeding. Furthermore, localized degradation of NETs by photoregulated release of DNase I abolished the NET-mediated capture and colonization of metastasizing colorectal cancer cells in the liver (103). Besides, NETs were also found to promote adhesion of tumor cells to distant organ sites by trapping circulating lung carcinoma cells within DNA webs, which further increased formation of hepatic metastasis (88). Inhibition of NETs attenuated the development of hepatic metastases, suggest that NETs were responsible for lung cancer metastasis. In another study, NETs could interact with, trap (CTCs), which further contributed to tumor metastasis in lung cancer *in vitro* and vivo (104).Moreover, both NETs and CTCs expressed β1-integrin protein, which acted as a bridge mediating the interactions between CTCs and NETs, then increased cancer cell adhesion to distant organs (104). These findings highlight the molecular mechanism by which NETs can trap CTCs *via* a protein–protein interaction. Whether NETs-derived proteins have other molecular mechanisms to protect CTCs from risks, such as anoikis and apoptosis, are still unclear now. Thus, it is important to explore the mechanism of CTCs adhesion to NETs, that might identify NETs as potential therapeutic targets. Recently, NETs were found to trap hepatocellular carcinoma cells, and trigger the cytotoxicity resistance, enhanced invasiveness and angiogenesis of the trapped HCC cells (78). Mechanically, NETs enhanced metastatic of the trapped HCC cells by activating TLR4/9-COX2 signaling, that induced an inflammatory response (78). Yang et al. (15) demonstrated that NET-DNA functions as a chemotactic factor to attract CTCs, then induces cancer cells migration, adhesion, and distant metastases in breast cancer. Further study revealed that NET-DNA interact with coiled-coil domain-containing 25 (CCDC25) to activate the ILK-β-parvin-RAC1-CDC42 pathway, which may further facilitate the metastasis of cancer cells (15). Furthermore, Xiao et al (24). found that the protease cathepsin C activates the PR3-IL-1β axis, induces NET formation, and contributed to the early stage of metastatic colonization in breast cancer lung metastasis. Similar studies have shown that complement 3 (C3) is increased in lung mesenchymal stromal cells, and C3-C3a receptor axis promotes neutrophil recruitment and NET formation, which facilitates breast cancer cell metastasis to the lungs (105). And this function of C3 in the regulation of NETs depends on Th2-drived IL-4/IL-13-STAT6 pathway (105). Taken together, these studies confirm that NETs promote cancer metastasis through regulating multiple steps of cancer metastasis.

### 3.3 NETs in tumor therapy resistance

**Figure 2:**
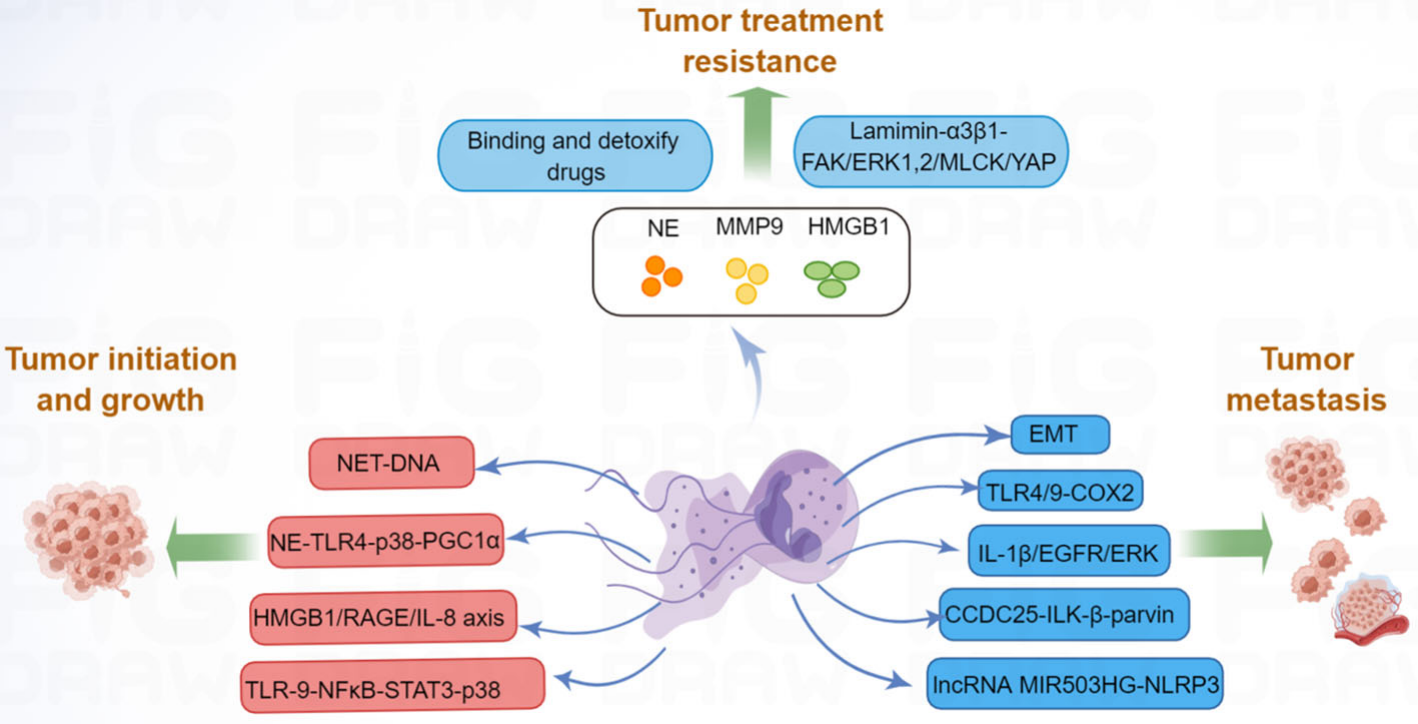
NETs promote tumor progression *via* many molecular pathways. NET can increase tumor cell proliferation by activating TLR9-NFκB-STAT3-p38 pathway; NET-DNA increased MMP-2 and -9 production, which increased tumor growth; NE released by NETs, can enhance tumor growth by activating TLR4-p38-PGC-1α pathway; HMGB1, released by NETs, can promote tumor growth by RAGE-IL-8 axis. Moreover, NETs promote tumor metastasis by promoting EMT, activating TLR4/9-COX2, IL-1β-EGFR-ERK, CCDC25-ILK-β-parvin, and lncRNA MIR503HG-NLRP3 pathway. Besides, NETs-associated NE, MMP-9, and HMGB1 contribute to tumor therapy resistance.

In addition to tumor growth and metastasis, tumor therapy resistance remains a major challenge in current research. Resistance to tumor includes both primary and secondary resistance. Targeted therapy is frequently associated with acquired resistance (106), whereas immunotherapy is often associated with primary resistance (107). In the area of malignancy, tumor-associated neutrophils (TANs) have been shown to contribute to cancer resistance to therapies (108). Building on the function of TANs in cancer resistance to therapy, NET-dependent mechanisms of drug resistance are beginning to be recognized. For example, drug-resistant cancer cells are dormant during clinical remission and can be reactivated leading to cancer recurrence (109). It has been demonstrated that NETs are required for awakening dormant cancer (110). Mechanistic analysis revealed that NET-associated NE and MMP-9 proteins cleave laminin and activate integrin α3β1 signaling, which further induces focal adhesion kinase (FAK), ERK1/2, myosin light-chain kinase (MLCK), and yes-associated protein (YAP) signaling to reactivate dormant cancer cell proliferation (110). Moreover, NETs could trap doxorubicin (DOX) and inhibit its diffusion into ovarian cancer cells; the degradation of NETs could increase the DOX-induced apoptosis of ovarian cancer cells (111), suggested that NETs induced DOX chemotherapy resistance. Radiotherapy is an important component of cancer treatment, however, radioresistance can lead to tumor progression and mortality (112). One study revealed that radiation therapy could stimulate NET formation in bladder cancer; in turn, increased NETs contributed to tumor radioresistance (87). Researchers further found that HMGB1 was released by tumor cells after radiation therapy, and HMGB1 promoted NET formation by activating TLR4 signaling (87). Inhibition of HMGB1 and NETs significantly delayed tumor proliferation. Moreover, NET levels were significantly higher in radiation therapy non-responders than in radiation therapy responders, suggesting that NETs seem to have a pivotal influence on radioresistance (87). Additionally, another study indicated that NETs participated in the post-radiotherapy local recurrence of in breast cancer (113). NETs are increased in relapsed human breast cancer and are associated with poor prognosis, and inhibition of NETs might provide new opportunities to address post-radiotherapy resistance in clinical trials. Overall, NETs play important roles in tumor progression (**Figure 2**), further research on the molecular mechanism of NET-mediated tumor progression is warranted.

## 4 NETs in immune cells

Beyond the well-known functions of NETs in the diversified phases of tumor metastasis and tumor progression, NETs also play critical roles in tumor immune exclusion. The tumor-promoting function of NETs is mediated not only by diverse mechanisms, as described above, but also by attenuating the antitumor functions of the immune system. Accumulating evidence suggests that NETs are considerably involved in the regulation of immune cells (114, 115).Thus, interest in understanding how NETs interact with immune cells to modulate the tumor immune response of tumors is increasing.

### 4.1 Macrophages and DCs

Macrophages and Dendritic Cells (DCs), two major Antigen Presenting Cells (APCs), are pivotal innate immune cells that regulate the anti-tumour immune responses (116, 117). It has been shown that NETs activate macrophages and DCs through upregulating important costimulatory molecules (CD80, CD86) at early times (30 min), however, macrophages and DCs undergo apoptosis after prolonged incubation with NETs (118). Further study showed that NETs-derived histone H2A and to a lesser degree elastase caused mitochondrial morphological alterations, which further induced a caspase- and AIF-dependent apoptosis (118).These results indicated that NETs interact with macrophages and DCs for a long time might enhance tumor immunosuppression. Another study revealed that LPS induced significant upregulation of surface markers of activation and maturation on DCs, such as, CD80, CD83, and CD86 was significantly reduced when DCs were exposed to both NETs and LPS (119). Moreover, NETs plus LPS significantly promoted inflammasome activation though increased IL-1β secretion, and decreased LPS-induced IL-10, an immunomodulatory cytokine, and IL-12, a T cell stimulatory factor in both macrophages and DCs (119). In turn, both macrophages and DCs could also degrade NETs (119), suggesting that NETs acted as double-edged swords of innate immunity. Besides, the addition of NETs to IL-4/GM-CSF-treated monocytes downregulated the expression of the IL-4 receptor in monocytes and prevented monocytes from fully differentiating into DCs but induced them to differentiate into M2 macrophages (120). It has been reported that M2 macrophages such as tumor-associated macrophages (TAMs) promote tumor growth and invasion (121). Thus, NETs might contribute to tumor progression through promoting M2 polarization of macrophages. Moreover, DNA released from NETs also mediated pro-inflammatory macrophage polarization by activating the TLR-9 pathway (122). In addition, NETs induced the production of IL-8 by macrophages through activating the TLR9/NF-κB signaling pathway, which further aggravated atherosclerosis (123). Georgakis et al. found NETs from patients with systemic lupus erythematosus activate plasmacytoid DCs (pDCs) to secrete IFN-α, correlating with severe, active disease (124). Mechanistically, immunocomplexes stimulated neutrophils release IL-33-decorated NETs, which recognized the IL-33 receptor ST2L on pDCs, and further activating TLR9-IRF7 pathway, leading to IFN-α secretion (124). Similarly, cigarette smoke extract-induced NETs also promoted pDCs maturation and activation (125). The role of pDCs in TME is still ambiguous now (126). Thus, we hold the opinion that whether NETs-mediated pDCs activation display active immunity functions or involved in immune tolerance is determined by the specific tumor microenvironmental. In contrast, another recent study demonstrated that NETs induced by oleic acid stimulated DCs caused increased levels of CD40, CD86, and human leukocyte antigen DR (HLA-DR), indicating that oleic acid-induced NETs facilitated the maturation and activation of DCs (40). NE is an important component of NETs. A recent study indicated that NE could impair macrophage phagocytic function through the cleavage of cell surface receptors or opsonins (127). Furthermore, treatment of immature DCs with NE induced the generation of CD4+FOXP3+Tregs, which showed suppressive activity *in vitro (* 128). NETs regulate macrophages and DCs through different pathways, indicating that NETs might be an important indicator for antitumor immune response.

### 4.2 Natural killer cells

Natural killer (NK) cells are an important subset of innate immune cells that are found to be essential for tumor immunosurveillance (129). One study showed that NETs might inhibit the function of NK cells by upregulating *LGAS9* and *CEACAM1* genes, which are negative regulators for NK cells in patients with COVID-19 (130). Other groups have confirmed that NETs can accumulate decidual NK cells, which leads to immunological disorders in the placenta in patients with systemic lupus erythematosus (131). Moreover, CG, an important component of NETs, cleaves the NK cell-associated activating receptor NKp46, which further impairs NK cell function, including IFN-γ production and cell degranulation (132), suggesting that NETs might inhibit NK-cell based antitumor response. In turn, NK cells also induced NET formation *via* IFN-γ secretion, which further promotes thrombus formation (133).

### 4.3 T cells

T cells have long been regarded as a major subset of the immune cells involved in tumor immunity. Miranda et al. demonstrated that Toxoplasma gondii-induced NETs promote CD4+ T cell recruitment and the secretion of IFN-γ, TNF, and IL-6, indicating that NETs contribute to the adaptive immune response (134). In addition, NET-stimulated DCs promote primary CD4+ T cell differentiation into T helper (Th) 1 and Th17 cells compared with DCs without stimulation by NETs (40). Consistent with this finding, it has been shown that NETs can directly promote naive T cell differentiation into Th17 cells (135). Further studies have shown that histones are involved in the NET-induced increase in Th17 cell differentiation, and this regulation is dependent on the TLR2/MyD88 pathway. Moreover, NETs could also activate Th17 cells, that enhanced immune cells recruitment in atherosclerotic plaques (136). These findings demonstrate that NETs may be acritical factor influencing the differentiation of Th17 cells. It has also been reported that increased infiltration of Th17 cells promoted tumor progression and was correlated with a poor prognosis (137, 138). By inducing Th17 cell differentiation, NETs might be important for Th17 cell-related cancer immunotherapy. Additionally, in patients with severe COVID-19, focal NETs were negatively associated with CD8+ T cell infiltration in lung tissues (139). Taken together, how to target NETs to improve Th helper-mediated anticancer immunity needs to be explored in the future.

### 4.4 B cells

**Figure 3:**
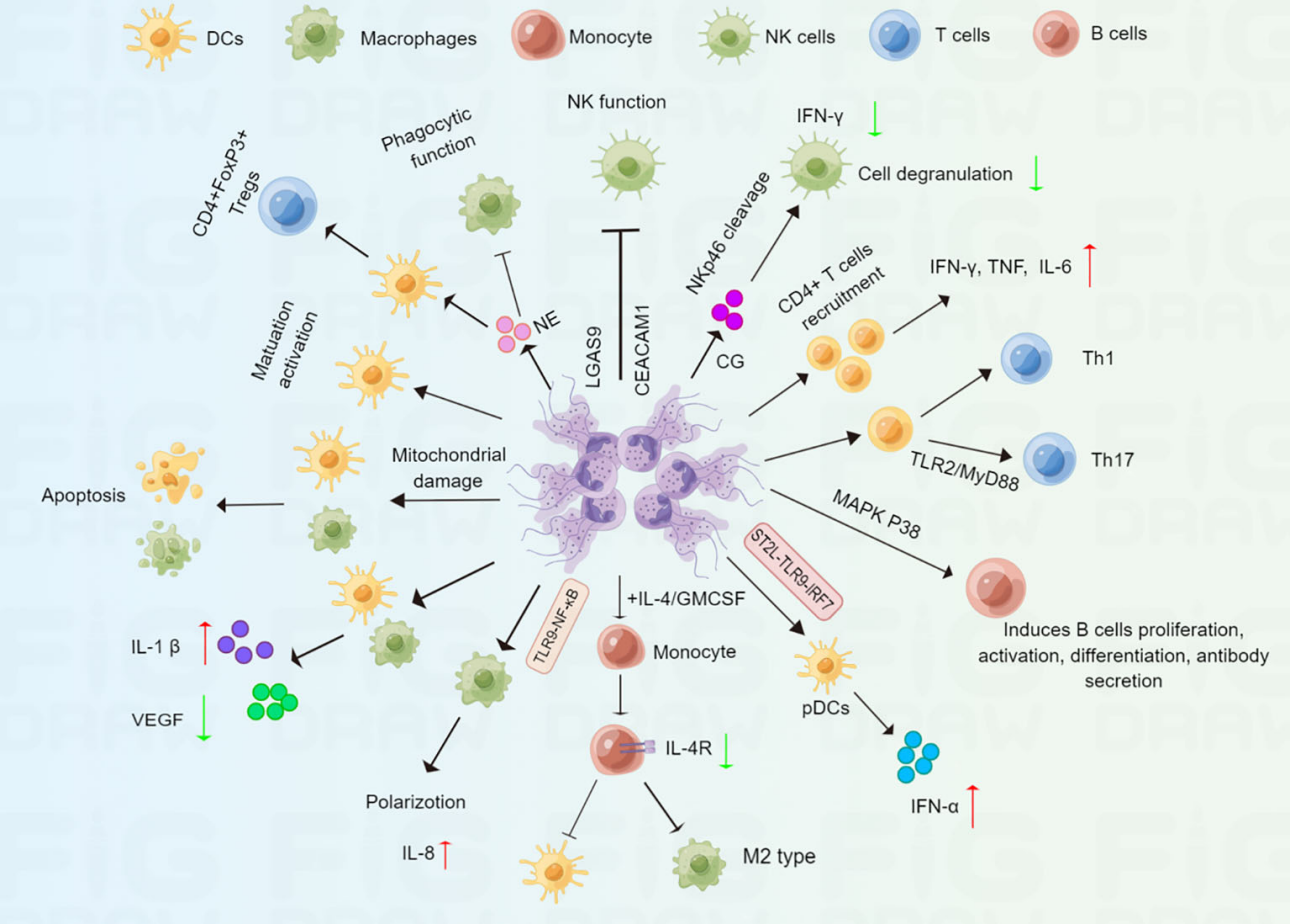
Schematic representation of NET in regulating immune cells. NETs can mediate immune response *via* complex regulations at multiple immune cells. Macrophages and DCs: NETs promote macrophages apoptosis, polarization, cytokine production, and impair macrophage phagocytic function; NETs can promote DCs apoptosis, maturation, activation and cytokine production. NK cells: NETs can impair NK cell function, including IFN-γ production and cell degranulation. T cells: NETs promote CD4+ T cell differentiation into Th1 and Th17 cell; NETs also promote immature DCs differentiation into CD4+FOXP3+Tregs.B cells: NETs can induce B cells proliferation, activation, differentiation and antibody secretion.

B cells could inhibit tumor progression through secreting immunoglobulins, promoting T cell response, and killing cancer cells (140). In addition to macrophages, DCs, and NK cells, NETs are also associated with B cells. For example, IL-37-DNA complexes derived from NETs can trigger B cell proliferation and activation in lupus erythematosus (LE) patients (141). Further study showed that NET-derived LL37–DNA complexes gain access to endosomal compartments of B cells and activate TLR9 pathway (141). In addition, citrullinated histones in NETs are thought to act as a continuous source of fresh antigens for B cells, promoting the production of new immunoglobulin M pathogenic anti‐citrullinated protein antibodies in rheumatoid arthritis (142).Another study showed that NETs might contribute to B cell activation and autoantibody secretion, which aggravates tissue damage in hidradenitis suppurativa (114). Moreover, elevated levels of NETs have been found to induce B-cell differentiation into plasma cells by activating the mitogen-activated protein kinases (MAPK) p38 pathway in bullous pemphigoid (143). These findings indicate that NETs might regulate tumor immune response. by acting on B cells. In summary, these studies suggest that NETs play an important but complicated role in immune cells (**Figure 3**).

## 5 Targeting NETs for tumor immunotherapy

Immunotherapy has provided new strategies for cancer therapy and has increased long-term survival in subsets of patients. The significant and wide-ranging effects of NETs in regulating tumor cells and immune cells have prompted the clinical investigation of additional therapies to improve the efficacy of tumor immunotherapy.

### 5.1 NETs in anti-tumor immunity

Given that there is much evidence for the participation of NETs in many types of immune cells, it is no surprise that NETs regulate tumor immunity. For instance, in non-small cell lung cancer, bladder cancer, and metastatic melanoma, NET density is inversely correlated with CD8+ T cell density, suggesting that NETs might impair CD8-mediated antitumor immunity (144). Furthermore, studies have shown that both CD4+ and CD8+ T cells in the NET-rich TME express significantly higher levels of T cell exhaustion-related markers, such as programmed cell death protein 1 (PD-1), T cell immunoglobulin domain and mucin domain 3 (Tim3), and lymphocyte-activation gene 3 (Lag3), indicating that increased NETs in the TME are responsible for the loss of T cell function (145). Further research demonstrated that both mouse and human neutrophil-derived NETs contained the immunosuppressive ligand programmed death-ligand 1 (PD-L1), blocking of PD-L1 in NETs obviously decreased tumor growth (145). In addition, NETs can obstruct contact between immune cells and the surrounding target tumor cells by wrapping and coating tumor cells and protecting them from CD8+ T cells and NK cell-mediated cytotoxicity, which further hamper immune-cell control of tumor metastases (23). Moreover, NETs inhibition sensitized tumors to PD-1+CTLA-4 dual checkpoint blockade (23). Another group reported that NETs participated in IL-17-associated immunosuppression in pancreatic cancer (20). Mechanistically, IL-17 recruited neutrophils, induced NETs formation, which favors tumor CD8+ T cell inactivation and spatial exclusion (20). Wang et al. recently demonstrated that NETs and regulatory T cells (Tregs) co-localized in NASH-associated HCC and that NETs could promote the differentiation of naïve CD4^+^ T cells into Tregs which contributes to the initiation and progression of NASH-HCC (146). Further study showed that NETs activated TLR4 pathway in naive CD4+ T cells, leading to naive CD4+ T cells metabolic processes reprogram, tilting the balance toward mitochondrial oxidative phosphorylation (OXPHOS) to promote Treg differentiation (146). In addition, another study demonstrated that NETs lead to a hypercoagulable state in gastric cancer (147). Further studies revealed that NETs upregulated angiopoietin-2 (ANGPT2), and ANGPT2 was significantly correlated with macrophage M0, NK cell resting, and mast cell activation, suggesting that NETs might be involved in the regulation of the immune microenvironment in gastric cancer. Other studies have shown that NET-related long intergenic non-protein coding RNA 426 (LINC00426) contributes to the innate immune cyclic GMP-AMP synthase (cGAS)-stimulator of interferon genes (STING) signaling pathway in head and neck squamous cell carcinoma (148). Taken together, these observations suggest that the pro-tumorigenic activities of NETs are also mediated by the attenuation of antitumor functions of the immune system, which occurs by impairing the function of tumor-antagonizing immune cells and the maintenance of an immunosuppressive molecular signature in the TME.

### 5.2 NETs in immunotherapy

As NETs are considerably involved in regulating the behavior of tumor cells and immune cells, thus affecting the efficacy of tumor immunotherapy in different ways. Therefore, targeting NETs is a relatively new option to inhibit tumor progression and boost the efficacy of immunotherapy, including decreasing NET formation and/or activity in tumors. Current trials targeting NETs are mainly based on interference with NETs formation or direct dismantling of their structure. For example, targeting of PAD4 with GSK484 inhibitor repressed NETs formation and prevented dormant cancer cell awakening in a breast cancer model (110); targeting PAD4 with the novel PAD4 inhibitor BMS-P5, delayed the appearance of symptoms and MM progression (149). In addition, targeting the tumor-associated induction of NETs formation is also a promising therapeutic strategy. ROS, TNF-α, IL-8, cathepsin C, amyloid β, and CXCR-1 and -2 are all responsible for NETs release, as mentioned above. Blocking these tumor-associated NET stimuli with antibodies or inhibitors might prevent metastatic colonization by abolishing NET-mediated capture of circulating tumor cells. Other groups have also focused on the interaction mediators present in NETs and cancer cells, such as integrin (104), TLR9 (94) and CCDC25 (15). Functional blocking of these mediators may also contribute to tumor treatment.

**Figure 4:**
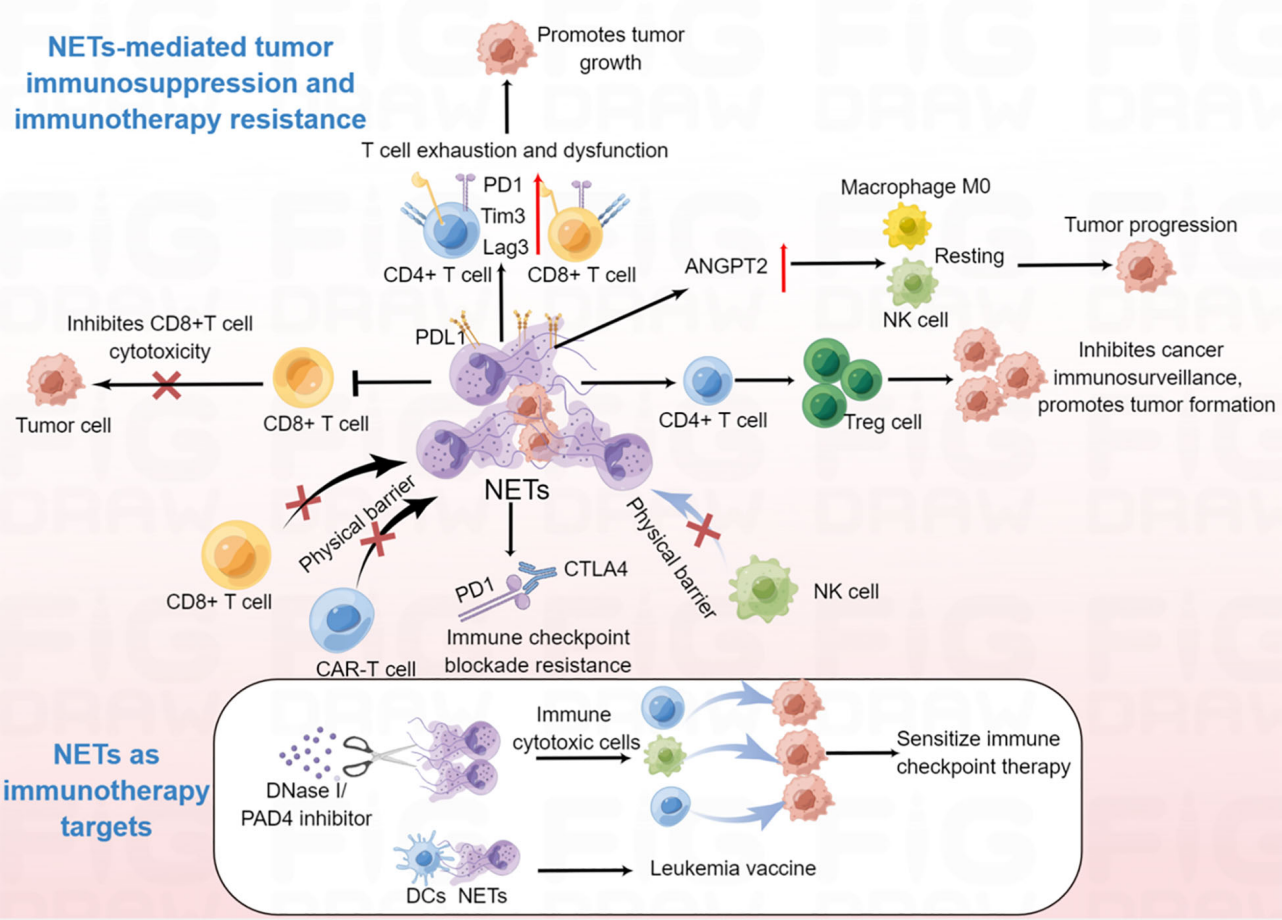
The emerging roles of NETs in the modulation of anti-tumor immunity and immunotherapy. NETs can promote CD4+ and CD8+ T cells exhaustion and dysfunction; NET-mediated physical barrier decreases the contact of immune cytotoxic cells (CD8+ T cell, NK cell and CAR-T cell) with tumor cells; NETs promote the differentiation of naïve CD4+ T cells into Tregs, which further contribute to tumor initiation and progression; NETs promote macrophage M0, NK cell resting. Degradation of NETs by DNase I can enhance the efficiency of tumor immunotherapy; NET/DC vaccine may be used for leukemia treatment.

Recent report has demonstrated that NETs are associated with immunotherapy resistance (150). NET-mediated physical barriers inhibited contact between immune cytotoxic cells and tumor cells and influenced immune checkpoint therapy in primary colorectal cancer (88). Using photoregulated enzyme delivery for efficient release of DNase I for localized degradation of NETs destroyed the NET-mediated physical barrier, thereby enhancing the interaction of immune cytotoxic cells with tumor cells, and sensitized immune checkpoint therapy for primary colorectal cancer, and eliminating NET-mediated capture and colonization of metastasizing cells in the liver sinusoids (88). These results suggest inhibition of NETs by DNase I facilitate the removal of immunosuppressive NETs, and improve the efficacy of clinical treatment. Similarly, high levels of NETs inhibited the response to anti-PD-1 therapy in a mouse colorectal cancer model (150). Furthermore, degradation of NETs by DNase I reduced tumor cell-induced TAN infiltration within tumors, and increased CD8+ T cell infiltration and cytotoxicity, which further improved the efficacy of PD-1 blockade to inhibit tumor growth (150). In addition, NETs also mediated resistance to immune checkpoint blockade PD-1 and cytotoxic T-lymphocyte associated protein 4 (CTLA4) by Ovarian cancer in pancreatic cancer (20). Besides, NETs could greatly counteract the efficacy of NK cell therapy and contribute to HCC recurrence (151). Inhibition of NETs enhanced NK cell infusion to kill cancer cells (151).These findings indicated that NET-mediated immunotherapy resistance is through protecting tumor cells from cytotoxic immune attack. Moreover, NET-associated T cell exhaustion was abrogated by DNase, which also supports the use of NET-targeting therapeutics to restore proper T cell antitumor activity. In addition, chimeric antigen receptor (CAR)-T therapy in solid tumors often resistance to immunotherapy, and NETs can prevent the interaction of CAR-T cells with tumor cells (152). Therefore, NET inhibition might overcome CAR-T resistance in the future. In addition, vaccination with DCs loaded with NETs reduced myeloproliferation in transgenic mice, and induced CD8+ T cell responses (153), suggesting that NETs might be used in the development of a leukemia vaccine. Taken together, NETs have the potential to enhance the efficacy of clinical immunotherapy by promoting T cell tumor infiltration and enhancing cytotoxic immune cells on tumor cells and could be used in tumor vaccines in the future (**Figure 4**).

## 6 Concluding remarks

While diverse studies have demonstrated the classic functions of NETs in promoting, tumor growth, metastatic spread and cancer therapy resistance, accumulating data in recent years have clearly shown that NETs play an important role in immune regulation. In this review, we summarized the functions of NETs in immune cells, anti-tumor immunity, and tumor immunotherapy. A better understanding of the crosstalk between NETs and anti-tumor immunity can help overcome cancer immunotherapy resistance. However, the role of NETs in anti-tumor immunity in other immune cells, including macrophages, DCs, myeloid-derived suppressor cells, B cells, and, has not been sufficiently evaluated. Moving forward, we believe that detailed analyses of the role of NETs in immune, tumor, and TME/stromal cells are required. Moreover, it should be noted that a number of proteins and potentially other NETs compounds may be detrimental for antitumor immune response. Thus, scientists need to carry out more research to identify the role of NETs-associated proteins in immunotherapy. These efforts would provide a substantial basis for targeting NETs as a new/alternative choice and a new approach for clinicians in cancer immunotherapy.

## Author contributions

MY: conception of the work, MY, YG, HS, and QG extensive literature search and manuscript drafting. MY and YG contributed to the editing and revising of this work. All authors contributed to the article and approved the submitted version.

## Glossary

**Table:**

ANGPT2	angiopoietin-2
C3	complement 3
CAR	chimeric antigen receptor
CCDC25	coiled-coil domain-containing 25
CG	cathepsin G
COVID-19	coronavirus disease 2019
CSF	colony stimulating factor
CTLA4	cytotoxic T-lymphocyte associated protein 4
CXCLs	C-X-C motif chemokine ligand
CXCR	C-X-C motif chemokine receptor
DC	dendritic cell
DOX	doxorubicin
ERK	extracellular signal−regulated kinase
GM	granulocyte-macrophage
H3Cit	citrullinated histone H3
HCC	hepatocellular carcinoma
HMGB-1	high mobility group box 1
IFN	interferon
IL	interleukin
MM	multiple myeloma
MMP-9	matrix metalloproteinase 9
MPO	myeloperoxidase
NASH	non-alcoholic steatohepatitis
NE	neutrophil elastase
NET	neutrophil extracellular trap
NF	nuclear factor
NK	natural killer
NOX	NADPH-oxidase
PAD4	peptidyl arginine deiminase 4
PD-1	programmed cell death protein 1
PD-L1	programmed death-ligand 1
PMA	phorbol 12-myristate 13-acetate
ROS	reactive oxygen species
TAN	tumor-associated neutrophil
Th	T helper
TLR	toll-like receptor
TME	tumor microenvironment
Tregs	regulatory T cells
TIMP1	tissue inhibitor of metalloproteinases-1
